# Glucose-methanol co-utilization in *Pichia pastoris* studied by metabolomics and instationary ^13^C flux analysis

**DOI:** 10.1186/1752-0509-7-17

**Published:** 2013-02-28

**Authors:** Joel Jordà, Camilo Suarez, Marc Carnicer, Angela ten Pierick, Joseph J Heijnen, Walter van Gulik, Pau Ferrer, Joan Albiol, Aljoscha Wahl

**Affiliations:** 1 Department of Chemical Engineering, Escola d’Enginyeria, Universitat Autònoma de Barcelona, Bellaterra, Cerdanyola del Vallès, Spain; 2 Department of Biotechnology, Kluyver Centre for Genomics of Industrial Fermentation, Delft University of Technology, Delft, The Netherlands; 3 Present address: Laboratoire Ingénierie des Systèmes Biologiques et des procédés, INSA, Toulouse, France

**Keywords:** *Pichia pastoris*, Instationary ^13^C-metabolic flux analysis, GC-MS, LC-MS

## Abstract

**Background:**

Several studies have shown that the utilization of mixed carbon feeds instead of methanol as sole carbon source is beneficial for protein production with the methylotrophic yeast *Pichia pastoris.* In particular, growth under mixed feed conditions appears to alleviate the metabolic burden related to stress responses triggered by protein overproduction and secretion. Yet, detailed analysis of the metabolome and fluxome under mixed carbon source metabolizing conditions are missing. To obtain a detailed flux distribution of central carbon metabolism, including the pentose phosphate pathway under methanol-glucose conditions, we have applied metabolomics and instationary ^13^C flux analysis in chemostat cultivations.

**Results:**

Instationary ^13^C-based metabolic flux analysis using GC-MS and LC-MS measurements in time allowed for an accurate mapping of metabolic fluxes of glycolysis, pentose phosphate and methanol assimilation pathways. Compared to previous results from NMR-derived stationary state labelling data (proteinogenic amino acids, METAFoR) more fluxes could be determined with higher accuracy. Furthermore, using a thermodynamic metabolic network analysis the metabolite measurements and metabolic flux directions were validated. Notably, the concentration of several metabolites of the upper glycolysis and pentose phosphate pathway increased under glucose-methanol feeding compared to the reference glucose conditions, indicating a shift in the thermodynamic driving forces. Conversely, the extracellular concentrations of all measured metabolites were lower compared with the corresponding exometabolome of glucose-grown *P. pastoris* cells.

The instationary ^13^C flux analysis resulted in fluxes comparable to previously obtained from NMR datasets of proteinogenic amino acids, but allowed several additional insights. Specifically, i) *in vivo* metabolic flux estimations were expanded to a larger metabolic network e.g. by including trehalose recycling, which accounted for about 1.5% of the glucose uptake rate; ii) the reversibility of glycolytic/gluconeogenesis, TCA cycle and pentose phosphate pathways reactions was estimated, revealing a significant gluconeogenic flux from the dihydroxyacetone phosphate/glyceraldehydes phosphate pool to glucose-6P. The origin of this finding could be carbon recycling from the methanol assimilatory pathway to the pentose phosphate pool. Additionally, high exchange fluxes of oxaloacetate with aspartate as well as malate indicated amino acid pool buffering and the activity of the malate/Asp shuttle; iii) the ratio of methanol oxidation vs utilization appeared to be lower (54 vs 79% assimilated methanol directly oxidized to CO_2_).

**Conclusions:**

In summary, the application of instationary ^13^C-based metabolic flux analysis to *P. pastoris* provides an experimental framework with improved capabilities to explore the regulation of the carbon and energy metabolism of this yeast, particularly for the case of methanol and multicarbon source metabolism.

## Background

*Pichia pastoris* has become an important yeast cell factory platform for the production of recombinant proteins [1-3] and has additional potential applications for whole cell biocatalysis [4,5]. Moreover, the development of systems biotechnology tools specific for this cell factory [6-11] has increased the knowledge for rational, model based strain improvements [12], as well as optimization of media composition and culture conditions. A prominent feature of the *P. pastoris* system is the use of the strong *AOX1* promoter from the alcohol oxidase 1. The conceptual basis for this expression system stems from the observation that some of the enzymes required for methanol metabolism are present at substantial levels only when cells are grown on methanol [13,14].

Mixed-carbon substrate feeding strategies (typically mixing methanol with a multi-carbon source such as sorbitol or glycerol) have been extensively investigated for *P. pastoris* based bioprocesses [15]. Such strategies have proven to boost recombinant protein production rates significantly during high cell density cultivations, suggesting an impact of recombinant protein production on the cell’s energy metabolism [16-19]. In fact, recent quantitative physiological studies have provided new insights on the metabolic burden derived from recombinant protein overexpression in yeast (*P. pastoris*) and fungi [9,20-22]. Most notably, all authors report that recombinant protein production caused a significant carbon fluxes redistribution, enabling increased supply of energy resources (NADH, NADPH, ATP) in the recombinant strains.

^13^C-based metabolic flux analysis has become a highly relevant tool for systems biology of microbial metabolism. Usually, ^13^C flux analysis relies on the detection of ^13^C patterns of proteinogenic amino acids using NMR or GC-MS. This approach requires long labelling phases as isotopic steady-state is required. Only then the enrichment of carbon backbones of key precursor metabolites is also present in the amino acids of the cell protein [23-26].

The direct measurement of isotopic enrichments of intracellular primary metabolites allows for shorter labelling experiments and additionally more information, and consequently a more accurate estimation of fluxes, including fluxes beyond central carbon metabolism [27,28]. However, fast and accurate quenching and metabolite extraction techniques are required for quantitative analysis of intracellular metabolites. Fortunately, a quenching and metabolite extraction method has been recently developed for *P. pastoris* cells growing on glucose [11], opening the door to metabolomics and metabolic flux analysis studies based on direct measurements of isotopic enrichments of the intracellular metabolites. In the present study, this metabolite quantification method has been validated for cells growing in the presence of methanol.

The use of more than one carbon source is also interesting in the context of ^13^C-based flux analysis methods. Currently, the approach is mostly used to determine steady-state fluxes in microbes grown on single-carbon substrates, typically glucose. Under single-carbon conditions, different approaches have been compared [29]. For co-assimilation of carbon sources, no comparison of results from different platforms is available. Moreover, because metabolic flux estimations could dependent on the applied technique, the combination of different ^13^C quantification techniques and mathematical formalisms may generate more accurate metabolic phenotype descriptions [30].

Recently, we extended the classic formalism for flux analysis of *P. pastoris* growing on glucose-methanol mixtures using ^13^C-labelling patterns of proteinogenic amino acids (measured by NMR) as constraints [22]. Nevertheless, this approach did not allow for an accurate mapping and quantitative characterisation of methanol metabolism and related pathways (particularly, for the case of multi-carbon source co-assimilation).

In addition, ^13^C-labelling information from primary metabolism has been used for the first time to estimate flux patterns of yeast cells growing on glucose-methanol substrate mixtures. Compared to previous studies [22], we could extend the metabolic network, and achieve new insights on the network topology and the reversibility of several metabolic reactions. In addition, the comparison of ^13^C-MFA based on the MS dataset allowed to validate the existing NMR-based methodological development for MFA.

Based on the obtained results, the cofactor balances were studied and novel hypotheses for the mixed substrate condition are derived.

## Methods

### Strain and cultivation conditions

A recombinant *P. pastoris* X-33 (Invitrogen) derived strain harbouring pGAPαA (Invitrogen) as a mock plasmid [31] was used throughout this study.

Duplicate chemostat cultivations were performed in a 2-l bioreactor (Applikon, The Netherlands) with a working volume of 1 l, using the Sartorious Biostat B + controller. The culture media were the same as described in [32]. In particular, the amount of C- and N-source of the chemostat medium was reduced to obtain a steady state biomass concentration of approximately 4 g/l. The glucose-to-methanol ratio was 4:1 [22]. All other components were adjusted accordingly to obtain similar residual concentrations comparable to previous cultivations. Also, the composition of the batch medium was adjusted according to the required biomass concentration, 8 g/l glycerol, 0.9 g/l citric acid monohydrate, 12.6 g/l (NH_4_)_2_HPO_4_, 0.5 g/l MgSO_4_.7H_2_O, 1.5 g/l KH_2_PO_4_, 0.02 g/l CaCl_2_ · 2H_2_O, 5 ml/l trace salt solution, 2 ml/l Biotin solution (0.2 g/l). The composition of the chemostat medium was 7.04 g/l Glucose monohydrate, 1.6 g/l Methanol, 0.915 g/l citric acid, 2 g/l (NH_4_)_2_ HPO_4_, 0.3 g/l MgSO_4_.7H_2_O, 1.4 g/l KH_2_PO_4_, 0.01 g/l CaCl_2_ · 2H_2_O, 0.5 ml/l trace salt solution, 0.3 ml/l biotin (0.2 g/l). The composition of the trace salts solution was the same as described previously [32].

A one litre shake flask containing 200 ml of YPD medium (10 g/l yeast extract, 20 g/l peptone, 10 g/l glucose) was inoculated with 1.5 ml of cryostock. The culture was grown for approximately 20–24 h at 30°C (shaking at 250 rpm), and used to inoculate the bioreactor. After termination of the batch phase (based on the CO_2_ profile, approximately 24 h after inoculation), the feed was switched on. Cells were growing under carbon limited conditions at a dilution rate of 0.09 h^-1^. The aeration rate was set to 1 vvm, controlled by mass flow meters (5850 Smart Mass Flow Controller, Brooks Instrument). The pO_2_ was maintained above 40% ensuring fully aerobic conditions. The O_2_ and CO_2_ concentrations in the bioreactor off-gas were measured on-line using a combined paramagnetic/infrared analyser (NGA 2000, Rosemount, USA). Pressure, pH, stirring speed and temperature were maintained at 1.2 bars, pH 5 (with 20% v/v NH_3_), 490 rpm and 25°C, respectively.

### Labelling experiment

After a minimum of five residence times of continuous cultivation, the feed was switched to the labelled medium. The composition of the labelling feed was based on the approach proposed by Nöh and Wiechert [33]. The feed contained for glucose, 80% [1-^13^C_1_] and 20% [U-^13^C_6_] and for methanol, 100% [U-^13^C_1_]. Immediately after switching to labelled medium feed, more than 10 samples were taken during the first five minutes; thereafter, 10 more samples were taken evenly distributed until 6 hours of labelling feed. The labelling experiment was performed in two independent chemostat cultivation replicates.

### Sampling and measurement of metabolite concentrations

Samples for intracellular metabolites and amino acid concentration measurements were taken one hour before the switch to the labelled feed using a dedicated rapid-sampling setup [34]. For quenching and extraction, the protocol recently described for *P. pastoris* growing on glucose [11] was used. Growth on methanol has been described to modify the cell wall composition of *P. pastoris*[35] – therefore, the protocol was re-validated using two different quenching liquid temperatures (90 and 95°C) and three different boiling times (5, 7 and 10 min) were tested, including the ones proposed by [11]. Since no significant differences in the metabolite pool concentrations were observed (data not shown) the original protocol of [11] was used during the experiment. Approximately 1 ± 0.01 g of broth were rapidly withdrawn and immediately mixed with 5 ml of precooled quenching solution (-40°C). For accurate metabolite quantification ID-MS (Isotope Dilution Mass Spectrometry) was applied [36,37], 120 μl of a ^13^C cell extract (4°C) were pipetted on top of the filter cell cake. The ^13^C cell extract contained all relevant metabolites as U-^13^C-labeled isotopes and was obtained from a *S. cerevisiae* fed-batch culture grown on 100% U-^13^C-labelled glucose and U-^13^C ethanol [36].

The metabolite extraction was performed with 75% (v/v) aqueous ethanol at 95°C. Concentrations were measured from two independent chemostat experiments in triplicate. The labelling enrichment was measured from one wash-in experiment by sampling at 20 time-points. The sampling times were selected based on the theoretical calculations by Nöh and co-workers [38], exponentially increasing intervals over a total of 6 hours to also achieve steady state in large intracellular pools like glutamate. The first sample is taken at 5 s after the switch to labelled material, which is the maximum velocity for manual sampling. All further sample processing steps were carried out as described previously [39]. Cell samples obtained during the labelling experiment were analysed by LC/MS and GC/MS [40]. The mass shifts of ^12^C samples compared to ^13^C-labelled cell extract were used to confirm the metabolite fragments. The obtained mass isotopomer distributions were corrected for derivatization-based as well as non-carbon isotope mass-shifts using the MS correction tool [41].

### ^13^C-based metabolic flux analysis (^13^C-MFA)

The metabolic network used for ^13^C-based metabolic flux analyses was derived from the metabolic model described in [22] for *P. pastoris*. As discussed in the Results section, this initial model was further extended to include trehalose metabolism and the pyruvate dehydrogenase bypass reactions, as well as reversible reactions for Ala, Asp and Glu synthesis. Additionally, an alternative stoichiometry of the pentose phosphate pathway (PPP) was included to evaluate the impact of the mechanisms proposed by Kleijn [42]. Because no statistically significant differences were obtained with the expanded PPP, the classical PPP stoichiometry was used (see also Additional file 1).

The expanded metabolic model established in this study contains 37 metabolites and 79 metabolic reactions (including bidirectional fluxes), (see metabolic model in Additional file 2). Also, seventeen independent fluxes were constrained based on:

1) the measured substrates and products uptake/secretion rates (three fluxes),

2) a series of biomass synthesis fluxes which were determined from the composition of the *P. pastoris* biomass and growth rate [22] (nine fluxes),

3) a set of fluxes that have symmetrical products (four fluxes, Met2, Tca4, Tca5 (fwd & bwd)) – a split of 1:1 for each form of the product was applied,

4) one constraint flux (emp11C = emp11D) for an unknown unlabelled pyruvate source (and sink). Source and sink were set equal to maintain the carbon balance.

**Figure 1:**
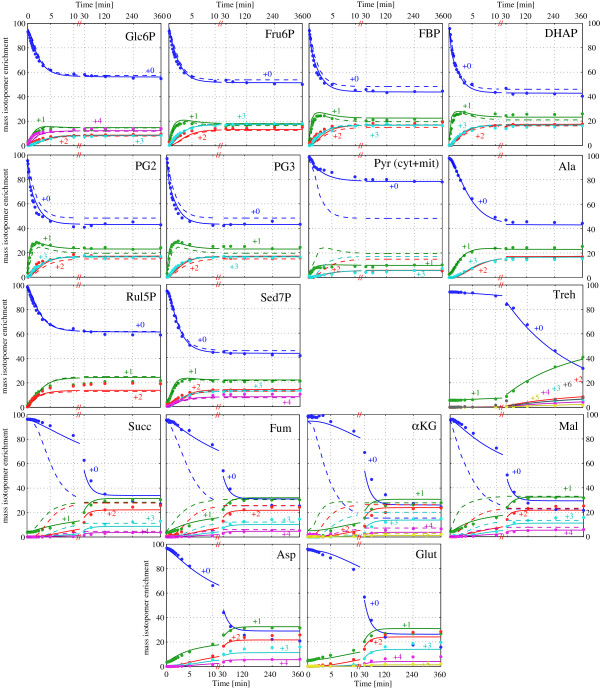
**Dynamics of mass isotopomers distribution of metabolites in *P. pastoris *chemostat cultures after switching to **^**13**^**C-labeled substrates.** Experimental data points are represented as solid circles. Solid lines reflect the simulation with the best flux estimation using the extended metabolic model (see Additional file 2). Dashed lines reflect the simulation with the best flux estimation using the metabolic model previously defined by [22].

Thus a total of 24 metabolic fluxes are required to solve the complete flux distribution. The mass isotopomer measurements, used for parameter estimation, are presented in Figure 1 and listed in Additional file 3. Of the 37 metabolite concentrations, required for the dynamic solution, a total of 27 were measured (see Additional file 4 for details), leaving 10 to be determined from parameter estimation.

For the simulation of the labelling enrichments, the instationary approach using the cumomer concept [27] was applied. The solution of the resulting system of ordinary differential equations and the parameter estimation procedure was implemented in gPROMS 3.1 (PSE Limited, London).

The parameter estimation routine implemented in gPROMS is based on maximum likelihood (SRQP iterative algorithm). The accuracy of the measurements was described by a constant relative variance, For mass isotopomer measurements a constant relative variance of 2% was used (based on previous experience).

For all results presented in this work a flux fit was considered acceptable when the obtained minimal weighted residual was below the *χ*^2^ at a 95% confidence level for the corresponding degrees of freedom. The degrees of freedom were calculated as the total number of experimental points (80 mass isotopomer fractions at 20 different times result in a total of 1600 data points) minus the number of parameters (34 in the expanded model). As a result of the fitting gPROMS provides the determined parameters together with the standard deviations and 95% confidence intervals (calculated based on error linearized error propagation).

### Network-embedded thermodynamic analysis

The consistency of the measured intracellular metabolite concentrations was verified with a method that is based on the second law of thermodynamics, A reaction can only run in the direction of a negative change of the Gibbs free energy [43]. This network based approach also allows to constrain the concentration of some unmeasured metabolites. All calculations were performed using the anNET software developed by Zamboni and co-workers [44]. Thermodynamic calculations are most conveniently performed using molar concentrations. For the conversion of the experimental results (μmol/g_CDW_), different values of cell volume were tested due to the lack of information of its real value for *P. pastoris* in the experimental conditions tested. Specifically, a range of cell volumes were tested, based on reported data for *S. cerevisiae*, ranging from 1.4 to 2.0 ml/g_CDW_[45]. However, the thermodynamically derived constrains obtained were equivalent, independently of the cell volume (data not shown). Therefore, the cell volume was fixed to 1.7 ml/g_CDW_ to perform all NET analysis. Cytosol was the only cellular compartment considered in this analysis, as no information on the specific volumes of the different compartments was available. Thus, the metabolic pathways included in the NET analysis and the intracellular metabolites were considered to be located in this compartment, including the methanol utilization reactions.

**Figure 2:**
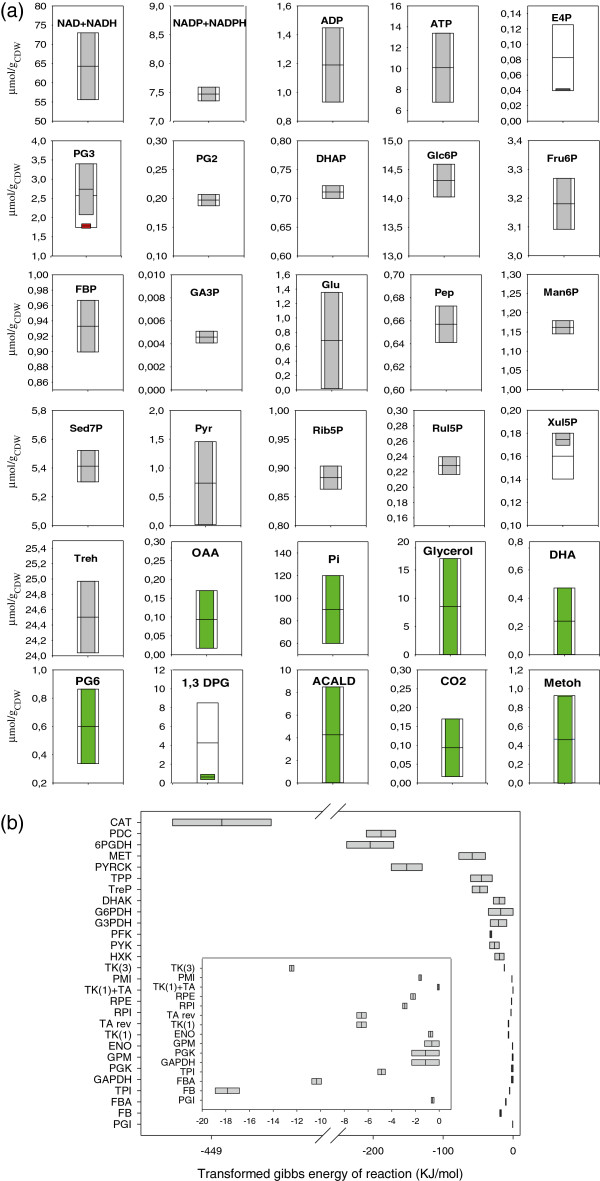
**Thermodynamic analysis of the *P. pastoris *reaction network, (a) Thermodynamically feasible concentration range of the measured metabolites and expected ranges of the non measured ones.** The white bars represent *a priori* considered metabolite ranges, the light grey bars (measured metabolites) and the green bars (unmeasured metabolites) show the corrected values after performing a network-embedded thermodynamic (NET) analysis. In case of detection of a significant metabolite quantification error, the original measurement (red bar) and concentration ranges before and after the NET analysis are shown together. **(b)** Transformed Gibbs energy of the cytosolic reactions of the central carbon metabolism of *P. pastoris* growing on glucose-methanol.

The feasible ranges of the quantified intracellular metabolites were calculated using the respective experimental mean and standard deviation. The lower and upper limits were defined according to a Student’s –T distribution with a confidence interval of 0.80. In addition, for the unmeasured metabolites ranges were defined (Figure 2a). From the NET analysis these were partly reduced to fulfil the thermodynamic constrains [44,45].

The assumed ionic strength of the *P. pastoris* cytosol was 0.15 M [46], while the pH was fixed to 7.2, based on experimental measurements of *P. pastoris* cytosol (D. Mattanovich, personal communication). This value is slightly higher than that reported for *S. cerevisiae*[47]. Nevertheless, the calculations were performed also at pH 7.0 and 7.5, globally obtaining equivalent results, except for the NAD^+^/NADH minimum ratio, as expected (see Results section).

### Analytical procedures

*Biomass Analyses,* The cell concentration was monitored by measuring the optical density of cultures at 600 nm (OD_600_). For cell dry weight (CDW) measurement, 5 ml of culture broth was filtered using pre-weighed dried glass fibre filters (Millipore). Cells were washed twice using the same volume of distilled water and dried overnight at 100°C. Triplicate samples (5 ml) were taken for all optical density and cell dry weight measurements. The macromolecular and elemental composition, used to derive the biomass equation for ^13^C-constrained metabolic flux analysis was previously obtained in cultures growing in analogous conditions as previously reported [22]. In all the chemostats the C recovery data was above 98% before applying a data consistency and reconciliation step. The experimental data was verified using standard data consistency and reconciliation procedures [48-50], under the constraint that the elemental conservation relations were satisfied. For all chemostat cultivations performed, the statistical consistency test was passed at a confidence level of 95%, and consequently there was no proof for gross measurement errors.

*Quantification of extracellular metabolites*, Triplicate samples (5 ml) for extracellular metabolite analyses were centrifuged at 6,000 rpm for 3 min in a micro centrifuge (Minispin, Eppendorf) to remove the cells, and subsequently filtered through 0.45 mm-filters (Millipore type HAWP). Glucose, methanol, and other extracellular compounds were analyzed by HPLC analysis using an ionic exchange column, (ICSep ICE-COREGEL 87H3, Transgenomic). The mobile phase was 6 mM sulphuric acid.

## Results and discussion

### Chemostat cultivation

*P*. *pastoris* cells were grown in aerobic, glucose-methanol limited chemostat cultures at a dilution rate of 0.09 h^-1^, resulting in comparable conditions to our previous study [22], except that cultivations in the present work were carried out at a lower cell density (~ 4.3 g/l). Under these conditions, biomass and carbon dioxide were the only products detected. Once the steady state was obtained (as reflected in constant macroscopic growth parameters over time), the consumption rates of glucose, methanol and oxygen and the production rates of biomass and carbon dioxide were calculated from measurements of biomass (cell dry weight, CDW), residual glucose and the concentration of oxygen and carbon dioxide in the off-gas line (Table 1).

**Table 1:** **Specific uptake and production rates of *P. pastoris *growing on glucose-methanol in chemostat cultures (D = 0.09 h**^**-1**^**)**

**Glucose (mmol/g**_**CDW**_** h)**	**Methanol (mmol/g**_**CDW **_**h)**	**OUR (mmol/g**_**CDW **_**h)**	**CER (mmol/g**_**CDW **_**h)**	**Biomass (mCmol/g**_**CDW**_** h)**	**RQ**
-0.71 ± 0.01	-0.94 ± 0.02	-2.57 ± 0.03	2.03 ± 0.03	3.14 ± 0.04	0.79 ± 0.04

Reconciled specific rates and calculated standard deviations [50] based on triplicate measurements; RQ, Respiratory Quotient. Consistency index h was below 5 for a redundancy of 3 (95% significance level).

### Metabolome analysis

**Figure 3:**
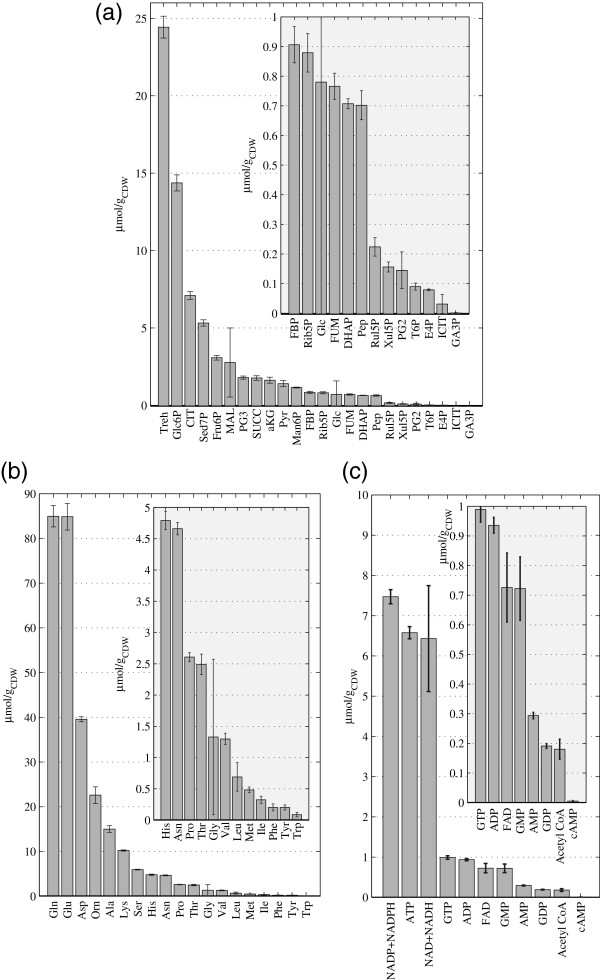
**Intracellular concentrations of *P. pastoris *cells growing on glucose-methanol. (a)** Central carbon intermediates **(b)**, Amino acids **(c)** Cometabolites and Nucleotides. Concentrations are given in μmol/g_CDW_.

In general, the intracellular levels of central carbon metabolites and free amino acids (Figures 3a to 3c, see also Additional file 4) were comparable to the values reported for *P*. *pastoris* cells grown on glucose as sole carbon source at the same growth rate [11]. Differences were observed for several metabolites of upper glycolysis – especially glucose-6-P (Glc6P), fructose-6-P (Fru6P) and fructose-1,6-P (FBP) – which were higher under glucose-methanol conditions. Interestingly, xylulose-5P (Xul5P) has a lower concentration in glucose-methanol grown cells indicating a clear influence of the methanol assimilation pathway activity.

Tricarboxylic acid (TCA) cycle intermediates and several metabolites from the pentose phosphate pathway (PPP) -Xul5P, erythrose-4-P (E4P) and sedoheptulose-7-P (Sed7P)- were higher under glucose-methanol conditions, indicating a shift in the thermodynamic driving forces.

As explained above, to validate the metabolite measurements, their thermodynamic consistency was checked using the Network-Embbeded Thermodynamic (NET) analysis described by [43,45], which is based on the principle that intracellular metabolite concentrations are constrained by the second law of thermodynamics, allowing specific directionalities of the metabolic reactions. For its application several assumptions were required, i) The most relevant metabolic fluxes are included in the central metabolism stoichiometric network described in [22], plus the fructose-1,6-bisphosphatase reaction (FBPase), ii) Peroxisome and cytosolic compartments are lumped into a single one, iii) concentration ranges for non-measured metabolites were constrained within thermodynamically feasible limits, and iv) the impact of sub-cellular compartmentation was ignored for co-factors.

Remarkably, concentrations of metabolic intermediates resulted to be thermodynamically consistent with the flux directionalities of the metabolic network reactions, with the exception of the metabolites involved in the non-oxidative branch of the pentose phosphate pathway (Figure 2a and Additional file 5). Thermodynamic consistency within this pathway could only be achieved by considering an alternative configuration for the transaldolase reaction (Figure 2b and Additional file 2). Such thermodynamic inconsistency has also been mentioned in other cases such as *S*. *cerevisiae*[51,52] and *P*. *pastoris*[53] growing on glucose. The inconsistency can be removed by including alternative considerations such as the introduction of a metabolite channelling step between the transketolase and transaldolase reactions (Xul5P + Rib5P ↔ Fru6P + E4P) allowed for a metabolic flux distribution consistent with the thermodynamic constrains derived from the intracellular metabolite levels, as also proposed for *S*. *cerevisiae*[52]. However, an inconsistency cannot be asserted in the present case taking into account the confidence intervals of the thermodynamic parameters and the fact that the PPP reactions operate close to the equilibrium.

The metabolite mass action ratios (MAR) of other reactions included in the central carbon metabolism and expected to operate close to equilibrium [45] were calculated (Table 2). Surprisingly, enolase (ENO) as well as phosphomannose isomerase (PMI) mass action ratios were found significantly different from the ones previously reported by [11,43]. The lower MAR calculated for PMI in this study compared with the equilibrium constant [54] indicates a lower capacity for this reaction step when a mixed carbon source is used.

**Table 2:** Comparison of mass action ratios of central carbon metabolism reactions under different cultivation conditions

***Enzyme***	***Mass action ratios***	***P. pastoris***		***S. cerevisiae***
		***Glucose-methanol***	***Glucose***^***a***^	***Glucose***^***b***^
PGI	Fru6P/Glc6P	0.22 ± 0.03	0.22 ± 0.11	0.26 ± 0.00
PMI	Man6P/Fru6P	0.36 ± 0.08	1.27 ± 0.06	1.18 ± 0.01
ENO	Pep/PG2	3.3 ± 0.29	1.67 ± 0.10	4.01 ± 0.09
FMH	MAL/FUM	3.7 ± 2.15	5.25 ± 0.38	5.15 ± 0.14

^a^Data taken from [11].

^b^Data taken from [40].

PGI, Phosphoglucose Isomerase, PMI, Phosphomannose Isomerase; ENO, Enolase; FMH, Fumarase.

The free amino acid pools measured for *P*. *pastoris* growing on the mixed carbon source were compared with those previously measured for the same strain growing with glucose as a sole carbon source. Although amino acids pools sizes show the same overall profile found in glucose-grown cells, generally lower amino acid pools sizes were found in cells grown on glucose-methanol. Nevertheless, such differences were only statistically significant (p-value < 0.05) for Tyr, Met, His, and Glu.

Strikingly, the extracellular concentrations of all measured metabolites were very low compared with the corresponding exometabolome of *P*. *pastoris* cells growing under analogous conditions but using glucose as sole carbon source. Exceptions are the residual glucose and, to a lesser extent, trehalose, which were significantly higher in the glucose-methanol cultures.

### ^13^C-Labelling dynamics and metabolic network extension

The dynamics of the measured mass isotopomer distributions are shown in Figure 1. As expected, the enrichment of the intermediates of TCA cycle and the storage carbohydrates such as trehalose were slower compared to glycolysis and PPP metabolites. All the glycolytic intermediates reached an isotopic steady state after about 15 min (< 5% variation from the enrichment at 4 h). Although fast, this time span is much higher than expected based on the metabolite concentration and the uptake rate (turnover time calculated for these metabolites). The delay has been observed consistently for eukaryotic systems and can mostly be explained by exchange fluxes with storage polymers (trehalose and glycogen) [55]. Besides this delay, additional unexpected patterns were found in the experimental data.

#### Glucose-6-phosphate (Glc6P)

The measurements of Glc6P are based on a fragment containing carbon atoms C3-C6. Two observations need to be highlighted for Glc6P, In contrast to other experimental conditions, the ^13^C enrichment in Glc6P is higher than expected from the direct precursor, extracellular glucose. Based on the feed composition (20% [U-^13^C_6_], 80% 1-^13^C glucose) the C3-C6 Glc6P fragment should be unlabelled (80%) resp. fully labelled (20%). Besides the m + 0 fraction, also the m + 1 (m + 2 and m + 3) fraction of this fragment shows unexpected values, especially by reaching a value of nearly 14% (10%, 8% resp.) at isotopic steady state. The origin of the masses +1, +2 and +3 is ^13^C carbon from methanol fixation. Especially DHAP shows a fast increase of the m + 1 mass fraction that via GA3P and the reversible pentose-phosphate pathway enters to Fru6P and finally Glc6P (reversible PGI reaction). Alternative to this route, the m + 1 (m + 2) can originate from metabolic activity of fructose-bisphosphatealdolase together with FBPase that can ‘re-scramble’ the C1 labelling into a C6 labelling of FBP (C1 labeled DAHP - > C3 labeled GA3P - > C1 or C6 labeled FBP). FBPase further carries this pattern to Fru6P [56]. Fru6P is very comparable to Glc6P indicating a high exchange flux of PGI.

Although the labelling dynamics of Glc6P are fast, an exchange with carbohydrate storage pools (here trehalose) needs to be introduced - Trehalose show a clear enrichment profile, that is faster compared to a pure synthesis flux (wash-in at *D* = 0.09 h^-1^), indicating a simultaneous synthesis and degradation of trehalose.

Based on these observations, trehalose synthesis and degradation reactions as well as FBPase were included into the metabolic network, reducing the difference between measured and simulated ^13^C-enrichment dynamics of upper glycolysis significantly (Figure 1).

#### Pyruvate

The enrichment of pyruvate is slower compared to its precursor PG2. It is commonly considered that alanine transaminase can slow down the pyruvate labelling due to the large pool size of alanine and the direct coupling with only one reaction step. However, our data clearly indicates that alanine is not the only source of the delay. The enrichment of alanine is actually faster than the enrichment increase measured in pyruvate. Therefore, it is assumed that the measured pyruvate labelling is a consequence of compartmentation (cytosol and mitochondria) together with additional, yet unknown exchange fluxes or pyruvate bypass reactions in the mitochondria. This is consistent with the finding that the other amino acids connected to the mitochondrial pyruvate node (valine, leucine, isoleucine) show a slower enrichment than alanine and (total) pyruvate enrichments.

Following the hypothesis of unknown exchange reactions, an unlabelled in-flux producing mitochondrial pyruvate was introduced together with a pyruvate efflux. Although this approach is not mechanistic, it allows to estimate the exchange with e.g. biomass components at this node [56]. This unlabelled carbon inflow into mitochondrial pyruvate, was estimated to be about 1.2 mmol/g_CDW_/h. Without this reaction, the measured enrichment of pyruvate could not be reproduced.

Note that this flux could also have an impact on the ATP balance, e.g. amino acid synthesis and degradation like the ones from the valine family would represent an ATP consuming cycle.

#### TCA cycle intermediates

The reproduction of the measured enrichments in the TCA cycle were improved by including:

1) Reactions for the pyruvate dehydrogenase (PDH) bypass (emp9 and emp12),

2) Exchange fluxes α–ketoglutarate ↔ Glu (aa_glu) and oxaloacetate ↔ Asp (aa_asp), that reflect a buffer capacity of amino-acid pools (e.g. large pools that are in exchange with low concentrated central carbon metabolites) and are maybe also involved in the activity of the Malate/Aspartate redox shuttle [57],

3) Asp ammonia lyase reaction which converts Asp into fumarate (tca8), resulting in NH_4_ formation and could reflect the activity of transamination reactions and cell protein turnover. This flux is not well determined as there is also a parallel route in the TCA cycle (tca7 and tca6).

Although the flux through the PDH bypass was not considered in the initial metabolic model (in fact, it was merged into the PDH reaction, [22]), its activity should not be excluded since Crabtree negative yeasts are reported to have PDH activity.

*S*. *cerevisiae* does not synthesise carnitine *de novo*, which is essential for carnitine acetyltransferase-mediated transport of cytosolic Acetyl-CoA (ACCoA) to the mitochondria. In contrast, a complete carnitine biosynthesis pathway has been characterised in *Candida albicans*, and the corresponding 4 genes have been identified [58]. Interestingly, the *P*. *pastoris* genome contains putative homologues to these genes [10,59] and proved to be transcriptionally active [60].

The TCA cycle metabolites do show a more pronounced delay to reach isotopic steady state (Figure 1). Besides reflecting the relatively slow labelling dynamics of glycolysis, carbon exchange due to transamination and protein turnover can significantly decrease the labelling enrichment speed as suggested by earlier simulation studies of transient ^13^C-labelling experiments in chemostat cultivations of *S*. *cerevisiae*[61].

#### Reversible reactions

Several glycolytic, non-oxidative pentose phosphate pathway and TCA cycle reactions, known to be reversible under physiological conditions, showed basically identical ^13^C-labelling transients between each of their corresponding substrate and product, suggesting high exchange fluxes. As discussed above, Fru6P and FBP labelling patterns suggested that both phosphofructokinase and fructose-1,6-bisphosphatase reactions were active in glucose-methanol grown cells. Therefore, the observed exchange reactions were also incorporated into the metabolic network model.

To analyse the impact of the network extensions and reversibility assumptions (resulting in the expanded model described in Additional file 2), we compare the simulated dynamics to a simulation based on the stoichiometric network defined in our previous study [22]. As observed in Figure 1 (dashed lines), that model did not allow for a consistent fit of simulated to measured isotopic enrichment dynamics. Furthermore, the expanded model allowed for a statistically acceptable fit based on the χ^2^ criteria as described in the material and methods section. More specifically, a total of 1600 experimental measurements (80 mass isotopomers * 20 time points) were available for the determination of 34 parameters, (24 fluxes and 10 concentrations). This results in 1566 degrees of freedom for the statistical χ^2^ test. The weighted residual of the best fit of the extended model was 1508.54 which is in the bounds of the chi-square distribution (1659.2, 95%), indicating an acceptable flux fitting.

In the case of the previous model, a weighted residual of 5540.2 was obtained for the best fit, which is above a 95% confidence level χ^2^ = 1239.3 with a total of 1160 experimental measurements (58 mass isotopomers * 20 time points) for the determination of 27 parameters, (17 fluxes and 10 unmeasured concentrations).

Thus, the newly added reactions were considered necessary to describe the behaviour of the yeast *P*. *pastoris* fed with mixed substrates. In the following steps, ^13^C-based MFA was performed using the LC-/GC-MS datasets and the expanded stoichiometric model (Additional file 2).

### Flux analysis results from instationary (INST) ^13^C-MFA

The simulation results for the two different models discussed previously, shown in Figure 1, together with the mass-isotopomer measurements obtained from LC- and GC-MS (Additional file 3), were further used to calculate the metabolic flux distribution through the central carbon metabolism of *P*. *pastoris* under glucose/methanol co-metabolism. The model partly considered compartmentation of metabolism. Specifically, for pyruvate a cytosolic and mitochondrial species were introduced, mainly motivated by the observations from a fast alanine enrichment, compared to a slow pyruvate enrichment. Although for the modelling, compartmentation is easily also introduced for other pools (including methanol assimilation peroxisomal reactions), the experimental results do only reflect whole cell amounts and labelling enrichments. Following our primary focus of determining fluxes of glycolysis, pentose-phosphate pathway and methanol assimilation we did not include complex, assumption based, approaches to fully include compartmentation. This simplification does not bias the estimation of fluxes in glycolysis, PPP or methanol utilization. Notably, quantification of labelling patterns of xylulose-5-P, dihydroxyacetone and glyceraldeyde phosphate, which are directly linked to methanol metabolism, could be measured by first time.

**Figure 4:**
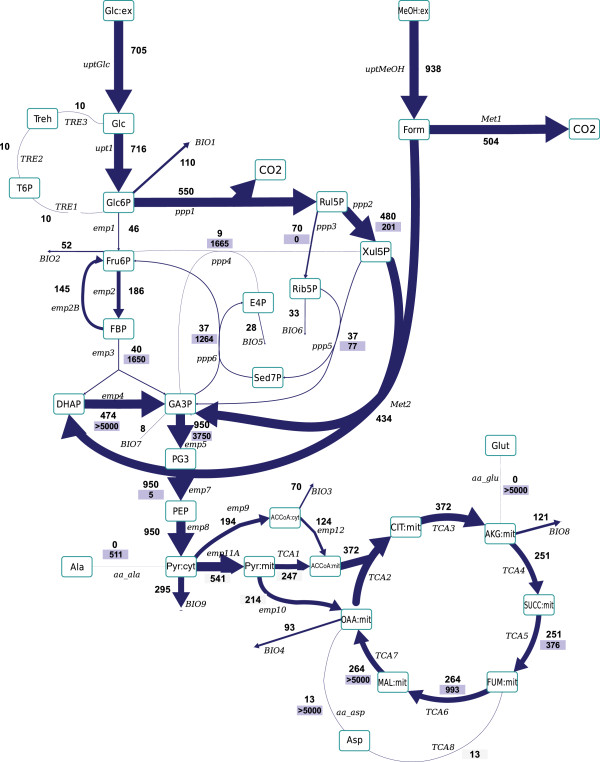
**Metabolic flux distribution based on **^**13**^**C flux analysis using the extended metabolic model.** The flux values are given in μmol/g_CDW_/h. The upper value represents the net flux (in direction of the arrow), the lower value (on blue background) reflects the backward flux (absolute). All fluxes are also listed in Additional file 6.

Generally, the overall carbon flux distribution pattern is similar to the corresponding flux pattern obtained using NMR data from ^13^C-labelling patterns of proteinogenic amino acids (Figure 4, see also Additional file 6), thus corroborating the mathematical formalism used to calculate metabolic flux ratios for the methanol assimilation pathways [22]. Specifically, following features of important network nodes are:

1) A major part of Glc6P is entering the oxidative branch of the PPP (55% compared to 57% calculated in our previous study using the NMR dataset);

2) Most assimilated methanol (54%) is directly oxidized to CO_2_ - lower than that calculated in our previous study (79%).

3) High exchange fluxes for oxaloacetate, malate, Asp and Glu, indicating amino acid pool buffering and the activity of a Malate/Aspartate shuttle (not determined previously).

4) Trehalose recycling accounts for about 1.5% of the glucose uptake rate (not determined previously).

The difference observed for methanol redistribution could be explained by the observed difference between the two experiments with respect to the specific methanol consumption rates (q_MetOH_). This rate was lower in the cultures performed in the present study compared with those from [22], (-0.94±0.02 mmol/g_CDW_/h *vs* -1.15±0.06 mmol/g_CDW_/h, respectively), resulting in a lower q_MetOH_/q_Gluc_ ratio. Since immediate oxidation of methanol to CO_2_ has also been considered a “detoxification pathway” to eliminate formaldehyde and formate, lower methanol consumption rates could result in lower relative activity of the dissimilatory pathway [62,63].

The high exchange fluxes around oxaloacetate, malate, Asp and Glu, could indicate an activity of the Malate/Aspartate shuttle [57]. This is coherent with previous NMR datasets from ^13^C-labelling experiments in *P*. *pastoris* growing aerobically on glucose or glucose-methanol [22,60], where the fraction of intact C2-C3 bonds of oxaloacetate were approximately equal for cytosolic and mitochondrial oxaloacetate pools. NMR data also indicated a high reversibility of the interconversion of cytosolic oxaloacetate to other cytosolic TCA cycle intermediates, further supporting the participation of Asp, oxaloacetate and malate in the redox shuttle for translocation of NADH across the mitochondrial membrane.

Although the metabolic fluxes around the pyruvate node still require further characterization, the MS data set clearly supports significant activity of the PDH bypass (about 30% of the mitochondrial ACCoA would be imported from the cytosol), which was not possible to discriminate using steady-state flux analysis based on ^13^C-NMR constrains.

Importantly, MS-based flux analysis provided novel insights regarding parallel resp. bidirectional reactions. In particular a gluconeogenic flux from dihydroxyacetone phosphate (DHAP) and glyceraldehyde phosphate (GA3P) back to Glc6P was observed based on the MS transient data. Moreover, such gluconeogenic flux resulted to be thermodynamically consistent with the measured glycolytic pool sizes (Figure 2a and 2b).

Also, by combining the metabolomic and MS-based flux analysis, we could more precisely determine the flux directionalities/reversibilities and stoichiometry in the non-oxidative branch of the PPP, which have been proven difficult to estimate accurately in previous studies on methanol co-assimilation [7]. Fluxes in the TCA cycle are less accurate as the transients after the pyruvate node are very slow. The flux estimation suggests high exchange fluxes in the TCA cycle based on very similar enrichment transients, but because of the relatively slow dynamics, the accuracy is lower (high standard deviations).

### Co-factor generation and consumption

Based on the ^13^C-based flux estimation (which only balances carbon and labelling), the co-factor balances are reconstructed using a stoichiometric model including the co-factors ATP, NADH, NADPH.

#### NADPH

As previously observed [22], methanol co-assimilation has a significant impact on the split ratio between the glycolytic and the oxidative branch of the pentose phosphate pathway. Substantially more carbon is channelled to the latter pathway compared to cells grown on glucose as sole carbon source. Consequently, NADPH regeneration flux generated from the oxidative branch of the PPP is higher in glucose-methanol than in glucose grown cells [53] (1.1± 0.1 *vs* 0.85±0.04 mmol/g_CDW_/h, respectively). In principle, since the PPP is the main pathway for cytosolic NADPH formation, the flux through the oxidative branch of the PPP is generally directly correlated to the biosynthetic demand for NADPH [64]. However, glucose-methanol co-assimilation does not result in increased biomass yields compared to growth on glucose as a sole carbon source [22]. This suggests that a surplus of NADPH may be generated. In fact, calculation of NADPH biosynthetic demand for cells grown on glucose-methanol was 0.97±0.07 mmol/g_CDW_/h. Although not statistically significant, this value was slightly lower than the total generated NADPH. In contrast, the NADPH biosynthetic demand for cells grown on glucose as sole carbon source exactly matched with NADPH generation. Interestingly, some Crabtree negative yeasts appear to have alternative mechanisms involved in the reoxidation of the NADPH produced in the PPP, e.g. by mitochondrial external alternative dehydrogenases that use NADPH [65]. Also, in *Pichia angusta* batch cultures growing on glucose, about 40% of the NADPH was produced in the PPP in excess of the biomass synthesis requirements, suggesting the presence of a yet unidentified NADPH reoxidation mechanism in this yeast [64]. It is tempting to speculate that this could also the case for *P*. *pastoris* cells co-assimilating methanol e.g. via the observed exchange fluxes between α-ketoglutarate and glutamate. Another potential sink for such excess of NADPH generated maybe related with the NADPH requirements for glutathione regeneration a key metabolite involved in the direct oxidation of methanol to CO_2_.

Additionally, protein turnover might have an impact here. The flux estimated for Asp lyase could indicate protein turnover and in its consequence NADPH consumption.

#### NADH

The rate of NADH regeneration was calculated from the reaction network stoichometry and calculated net fluxes as 4.37 ± 3.62 mmol/g_CDW_/h, i.e. within the range of the values previously calculated from the ^13^C-NMR based metabolic flux analysis [22]. Assuming that all NADH reduction equivalents, not consumed within the network, were recycled through the respiratory chain, the theoretical oxygen consumption rates were calculated. These represented about 92% of the measured q_O2_ - no significant imbalance that would suggest major NADPH dehydrogenase or NADPH → NADH transhydrogenase activities.

Strikingly, the NAD^+^/NADH minimal ratio calculated on the basis of the thermodynamic analysis of the metabolome data using the anNET software was about 1300 (assuming an intracellular pH of 7.2), which is over 13-fold higher than the one calculated for *P*. *pastoris* growing on glucose under equivalent conditions [53], (Table 3). This suggested that NADH generated by the action of formaldehyde and formate dehydrogenases in the cytosol is efficiently reoxidised, either by an NADH dedydrogenase located in the outer face of the mitochondria and/or transported to the mitochondria by means of a redox shuttle, as suggested above.

**Table 3:** Estimated NAD/NADH ratio under different cultivation conditions

	**pH (intracellular)**		
**Substrate**	**7.0**	**7.2**	**7.5**
Methanol-glucose	1906	1287	697
Glucose^a^	233	101	28

^a^Data taken from [53].

Reported are the minimal NAD^+^/NADH ratios (NAD^+^/NADH_min_), that fulfill the thermodynamic constraints (reaction directionality) at the measured and assumed concentrations (see Results section on Metabolome Analysis) for different intracellular pH.

#### ATP

The metabolic flux distributions calculated above allow to estimate the amounts of ATP generated or consumed either for growth or maintenance. Thus the total specific rate of ATP being generated by *P*. *pastoris* can be calculated as 8.6 ± 3.6 mmolATP/g_CDW_/h. Part of this ATP was generated by substrate level phosphorylation while the rest was generated by oxidative phosphorylation (respiration). Both terms can be calculated from the reaction stoichiometry and the net flux data. As a result, the fraction of ATP generated through respiration was about 75% of the total ATP generated

The maintenance requirements (non growth associated maintenance energy) was estimated to be 2.7 ± 3.6 mmolATP/g_CDW_/h. The efficiency of oxidative phosphorylation, i.e. the P/O ratio, was defined as 1.48 mol ATP/molO based on previous studies in yeast [66]. Considering the same P/O ratio, Chung and co-workers [8] reported maintenance requirements of 2.3 and about 6 mmol ATP/g_CDW_/h for *P*. *pastoris* growing on glucose and glycerol-methanol mixed media, respectively. These data can be compared with similar estimations from literature. Recent *P*. *pastoris* calculations based on a genome-scale model, [67] reported the ATP requirements for biomass synthesis to be strongly dependent on the substrate. In this case a global ATP generation range of 70–76 mmolATP/g_CDW_ was calculated for glucose and glycerol and a significantly higher one for methanol (ca. 150 mmolATP/g_CDW_). Mixtures of glycerol with methanol showed intermediate values. Based on these calculations, the specific ATP generation rates for *P*. *pastoris* cells growing in a chemostat at a *D* of 0.1 h^-1^ can be calculated to be in the range between 7 and 15 mmolATP/g_CDW_/h for glucose and methanol respectively, a range that includes the value of 8.6 mmolATP/g_CDW_/h calculated for the present case. Therefore, from a general point of view the above calculated energetic features of *P*. *pastoris* grown in chemostats using different combinations of glucose/methanol as carbon source are in agreement with previous estimates for yeasts.

## Conclusions

In this study, an MS-based INST ^13^C-MFA approach has been applied for the first time to *P*. *pastoris* co-assimilating glucose and methanol. Most notably, the flux distributions obtained with this methodology were coherent with those previously determined using ^13^C-labelling data of proteinogenic amino acids [22]. By using an INST ^13^C-MFA methodology, the *in vivo* metabolic flux estimations were expanded to a larger metabolic network and, reversibility of glycolytic/gluconeogenesis, TCA cycle and non-oxidative PPP reactions were determined. Besides, this approach allowed an improved accuracy for the calculation of fluxes through methanol assimilating pathways, as labelling patterns of some metabolite intermediates of these pathways (DHAP, GA3P_per_, PPP intermediates) could be directly accessed.

Combination of GC-MS and NMR datasets and additional ^13^C-labelling information from metabolites involved in compartment-specific reactions (used as “sensor reactions”) should provide improved accuracy to the current INST ^13^C-MFA approach, as well as providing new insights on the redox and energy metabolism. Especially the pyruvate node remains challenging and requires for an yet unknown exchange flux. This missing knowledge hampers also the precise reproduction of the TCA cycle derived amino acids, aspartate and glutamate at later time-points. Nevertheless, this does not influence the flux estimation of glycolysis and pentose-phosphate pathway because there is no exchange with these pools.

Combined analysis of metabolome and fluxome data showed their thermodynamic consistency, further validating the obtained results. The estimated flux directionalities were thermodynamically consistent when an alternative topology for the non-oxidative branch of the PPP (metabolite channeling between transketolase and transaldolase reactions) was used.

The surplus of NADPH generation, together with the gap in the NADH balance as well as increased ATP requirements for maintenance under glucose-methanol conditions could be an indication of increased protein turnover. Specific experiments, e.g. using ^15^ N could be applied to further investigate the cellular physiology under these conditions.

## Abbreviations

Metabolites

αKG: α-ketoglutarate; GA3P: Glyceraldehyde-3-phosphate; PG2: 2-phosphoglycerate; PG3: 3-phosphoglycerate; GA3Pper: Glyceraldehyde-3-phosphate peroxisome pool; DHA: Dihydroxyacetone; DHAP: Dihydroxyacetone phosphate; E4P: Eritrose-4-phosphate; Rib5P: Ribose-5-phosphate; Rul5P: Ribulose-5-phosphate; Xul5P: Xylulose-5-phosphate; Man6P: Manose-6-phosphate; Fru6P: Fructose-6-phosphate; Glc6P: Glucose-6-phosphate; Sed7P: Sedoheptulose-7-phosphate; FBP: Fructose-1,6-biphosphate; T6P: Trehalose-6-phosphate; Treh: Trehalose; Pyr: Pyruvate; Glc: Glucose; Pep: Phosphoenolpyruvate; MAL: Malate; SUCC: Succinate; CIT: Citrate; ICIT: Isocitrate; FUM: Fumarate; ACCoA: Acetyl coenzyme; Glux: Glucose intra/extra; FullyGlu: Fully labelled Glucose; CGlu: First carbon labelled Glucose; Metoh: Methanol; MetohL: Labelled Methanol; Xbio: Biomass formation; NPyr: Not labelled Pyruvate; Pyrt: Total Pyruvate pool; Form: Formaldehyde; FOR: Formate; OAA: Oxaloacetate; ACALD: Acetalehyde; PRPP: Phosphoribosyl pirophosphate; MTHF: Methylenetetrahydrofolate; THF: Tetrahydrofolate; CHOR: Chorismate; Pi: Inorganic phosphate; PPi: Inorganic pyrophosphate; 13dpg: 1,3 Diphophoglycerate; G1P: Glucose 1-phosphate; Glyc3P: Glycerol 3-phosphate; SUCCoA: Succinyl coenzyme A; Kval: Ketovalerate; DHF: Dihydropholate.

Other

nd: not determined; sd: standard deviation.

## Competing interests

The authors declare that they have no competing interests.

## Authors’ contributions

JJ performed bioreactor cultivations and ^13^C-labelling experiments, macroscopic data processing and metabolic flux analysis calculations. CS assisted in the bioreactor cultivations and the ^13^C extract preparation. MC assisted in the bioreactor cultivations and performed the thermodynamic analysis of metabolite datasets with the anNET programme. JJ together with AP, performed the concentration and ^13^C metabolite analyses. JJ, AW and JA extended the metabolic network, performed flux estimations and interpreted the results. PF, JA, JJH, WG participated in the overall conceptual and experimental design, interpretation of results and drafted the manuscript. All authors read and approved the final manuscript.

## Supplementary Material

Additional file 1 **Comparison of the ‘classical’ and the PPP stoichiometry proposed by Kleijn and co-workers **[42]**.** Click here for file

Additional file 2 **Metabolic models of the central carbon metabolism of *P*. *pastoris*. (1.1)** Reactions in the expanded stoichiometric model of the central carbon metabolism of *P*. *pastoris*, applied in the ^13^C-MFA. Note that CO_2_, O_2_ and cofactors were not used for flux balancing. (**1.2**) Reaction network used for anNET analysis. (**1.3**) Reaction- and atom transition network used in ^13^C flux analysis, following the notation of [68]. Click here for file

Additional file 3 Measured mass isotopomer fractions during the wash-in experiment. Click here for file

Additional file 4 **Measured extra- and intracellular concentrations at steady-state (D = 0.09 h**^**-1**^**).** Click here for file

Additional file 5 **Tables of the all Intracellular concentrations and Gibbs energy in *P*. *pastoris* cells growing on glucose-methanol chemostat.** Click here for file

Additional file 6 **Results from the INST **^**13**^**C flux analysis.** Estimated intracellular fluxes under methanol-glucose condition with the calculated standard deviations. The network description can be found in Additional file 2 **(1.1)**. Click here for file
