# Supplementary material for: Glucose-methanol co-utilization in Pichia pastoris studied by metabolomics and instationary 13C flux analysis
Source: BMC Syst Biol. 2013 Feb 28;7:17. doi: 10.1186/1752-0509-7-17 (PMC3626722; doi:10.1186/1752-0509-7-17)

**Additional file 1.**

- Classical reactions for the nonoxidative branch of the PPP (used in this study) consist of three reversible reactions, namely two transketolase (TK) catalysed reactions (r.1 and r.2) and one transaldolase (TA) catalysed reaction (r.3).

$Xul5P+R5P\leftrightarrow^{TK} S7P+GA3P$ r.1

$Xul5P+E4P\leftrightarrow^{TK} Fru6P+GA3P$ r.2

$S7P+GA3P\leftrightarrow^{TA} Fru6P+E4P$ r.3

- kleijn model, the structure of the traditional reactions is such a C2 or C3 fragment is transferred from one specific donor to one specific acceptor molecule. The nonoxidative branch of the PPP can be represented as metabolite specific, reversible C2 and C3 fragments producing and consuming half-reactions for each of the metabolite S7P, Fru6P, Xul5P, R5P, E4P and GA3P (r.4 to r.8)

$Xul5P\leftrightarrow^{TK} GA3P+E-C2$ r.4

$Fru6P\leftrightarrow^{TK} E4P+E-C2$ r.5

$S7P\leftrightarrow^{TK} R5P+E-C2$ r.6

$Fru6P\leftrightarrow^{TA} GA3P+E-C3$ r.7

$S7P\leftrightarrow^{TA} E4P+E-C3$ r.8

The next figure shows the comparison between both stoichiometry for de nonoxidative branch of the PPP. In solid line it has been represented the classical reactions and the dashed lines are represented the Kleijn model.


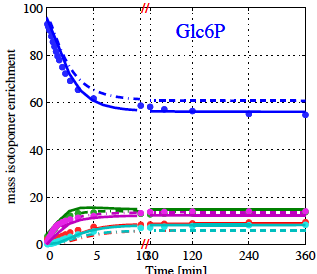

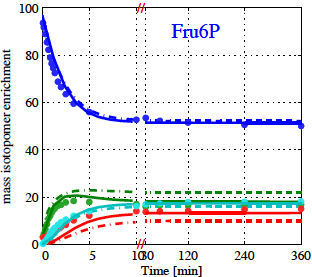

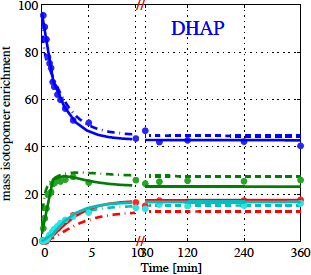

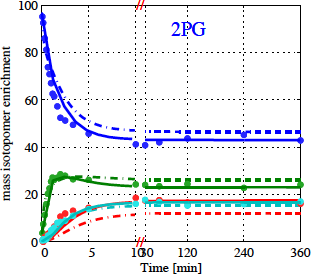

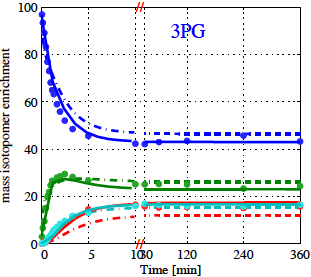

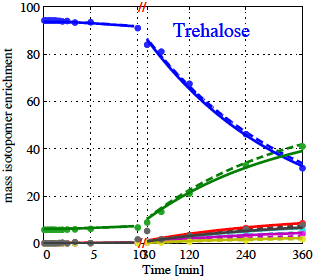

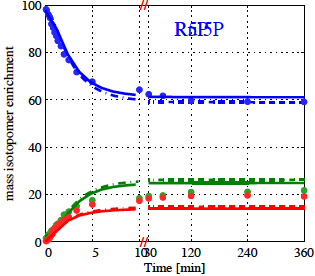

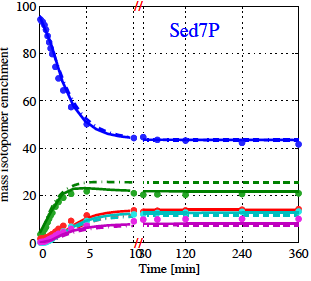

Supplement: Additional file 1 — Comparison of the ‘classical’ and the PPP stoichiometry proposed by Kleijn and co-workers [42]. [file 1752-0509-7-17-S1.docx]
