# Supplementary material for: Glucose-methanol co-utilization in Pichia pastoris studied by metabolomics and instationary 13C flux analysis
Source: BMC Syst Biol. 2013 Feb 28;7:17. doi: 10.1186/1752-0509-7-17 (PMC3626722; doi:10.1186/1752-0509-7-17)
Supplement: Additional file 2 — Metabolic models of the central carbon metabolism of P. pastoris. (1.1) Reactions in the expanded stoichiometric model of the central carbon metabolism of P. pastoris, applied in the 13C-MFA. Note that CO2, O2 and cofactors were not used for flux balancing. (1.2) Reaction network used for anNET analysis. (1.3) Reaction- and atom transition network used in 13C flux analysis, following the notation of [68]. [file 1752-0509-7-17-S2.doc]

**Additional file 2.**

**1.1 Stochiometric model for *P. pastoris* containing some additional reactions from the ^13^C model (section 1.2)**

**Methanol metabolism**

1. Metoh => Form
2. Form => FOR + NADH
3. FOR + NAD^+^ => NADH + CO_2_
4. Xul5P + FOR + ATP => ADP + GA3P_per_ + DHA
5. GA3P_per_ => GA3P
6. DHA => GA3P

**Glycolysis and gluconeogenesis pathways**

1. Glc_ext_ + 2 ATP <=> Glc6P + 2 ADP
2. Glc6P <=> Fru6P
3. Fru6P + 1 ATP => FBP + 1ADP
4. FBP => Fru6P + Pi
5. FBP <=> DHAP + GA3P
6. GA3P + ADP + Pi + NAD^+^ => PG3 + ATP + NADH
7. PG3 + ATP + NADH => GA3P + ADP + Pi + NAD^+^
8. PG3 <=> Pep
9. Pep + ADP => Pyr + ATP
10. Pyr + NAD^+^ => ACCoA_mit_ + CO_2_ + NADH
11. Pyr NAD ^+^ => ACCoA_cyt_ + CO_2_ + NADH
12. Pyr + CO_2_ + ADP => OAA + ATP
13. 2 Glc6P + UTP + H_2_O => H^+^ +2 Pi + UDP + T6P
14. T6P + H_2_O => Pi + Treh
15. Treh + H_2_O => 2 Glc6P

**Pentose phosphate pathway**

1. Glc6P + 2 NADP^+^ => Rul5P + 2 NADPH + CO_2_
2. Rul5P <=> Rib5P
3. Rul5P <=> Xul5P
4. Rib5P + Xul5P <=> Sed7P + GA3P
5. Sed7P + GA3P <=> Fru6P + E4P
6. Xul5P + E4P <=> Fru6P + GA3P

**TCA cycle**

1. ACCoA_mit_ + OAA => CIT
2. CIT => ICIT
3. ICIT + NAD^+^ => αKG + CO_2_ + NADH
4. αKG + NAD^+^ => SUCCoA + CO_2_ + NADH
5. SUCCoA + Pi + ADP => SUCC + ATP
6. SUCC + ATP => SUCCoA + ADP + Pi
7. SUCC + NAD^+^ => FUM + NADH
8. FUM + H_2_O <=> MAL
9. MAL + NAD ^+^ <=> OAA + NADH
10. Asp + 2 ATP + H_2_O=> FUM + 2 ADP + 2Pi

**Biosynthesis of amino acids**

**Serine family**

1. PG3 + Glut + NAD^+^ => Ser + αKG + NADH +Pi
2. Ser + THF => Gly + MetTHF
3. Ser + ACCoA + H_2_S => Cys

**Alanine family**

1. Pyr + NADPH => Ala + NADP**^+^**
2. Pyr + Glut => αKG
3. 2 Pyr + NADPH => Kval + CO_2_ + NADP^+^
4. Kval + Glut => Val + αKG
5. Kval + ACCoA_mit_ + Glut + NAD ^+^ => Leu + αKG + NADH + CO_2_

**Histidine family**

1. Rib5P + ATP => PRPP + AMP
2. PRPP + ATP + Gln + 2 NAD^+^ => His + αKG + Pi + 2 NADH

**Aspartic family**

1. OAA + Glut => Asp + αKG
2. Asp + Gln + ATP => Asn + Glut + AMP
3. Asp + ATP + 2 NADPH => Ser + ADP + Pi + 2 NADP^+^
4. Thr + NADPH + Glut + Pyr => Ile + αKG + NH_4_ + CO_2_ + NADP^+^
5. ACCoA + Ser + H_2_S + MTHF => Met + THF

**Aromatic family**

1. 2 Pep + E4P + ATP + NADPH => CHOR + ADP + 4 Pi
2. CHOR + Glut => Phe + αKG + CO_2_
3. CHOR + Glut => Tyr + αKG + NADH + CO_2_
4. CHOR + Gln + PRPP + Ser => Trp + Glut + Pyr + GA3P + CO_2_

**Glutamic family**

1. αKG +NH_4_ + NADPH => Glut + NADP + H_2_O
2. Glut + ATP + NH_4_ => Gln + ADP + Pi
3. Glut + ATP + 2 NADPH => Pro + ADP + Pi
4. Gln + CO_2_ + 2 ATP => CaP + Glut + 2 ADP + Pi
5. Glut + ACCoA_mit_ + 4 ATP + NADPH + CaP + Asp => Arg + αKG + 4 ADP + FUM + 5 Pi
6. 2 Glut + ACCoA_mit_+ 3 ATP + 2 NADPH + 2 NAD^+^ => Lys + αKG + CO_2_ + 2 NADH + 2 NADP^+^

**Biosynthesis and interconversion of one-carbon units**

1. DHF + NADPH => THF + NADP^+^
2. Gly + THF + NAD^+^ => MTHF + NH_4_^+^ + NADH + CO_2_
3. MTHF + NADH => THF + NAD^+^

**Transport reactions**

1. ACCoA_cyt_ => ACCoA_mit_
2. NH_4_^+^_ext_ + ATP => NH_4_^+^_cyt_ + ADP + Pi
3. SO_4_^-2^ _ext_ + ATP => SO_4_^-2^_cyt_ + ADP + Pi
4. Metal_ext_ + ATP => Metal_cyt_ + ADP + Pi

**Respiratory chain**

1. NADH + 0.5 O_2_ => NAD^+^ + H_2_O
2. ADP + Pi => ATP

**Biomass synthesis**

**1. Protein synthesis** (Composition derived from the measured amino acid composition [21]. The energy needed to biosynthesize 1 C-mol of protein was derived from the synthesis of each amino acid and the protein polymerization value taken from [50])

1. 0.148 Pyr + 0.083 NAD^+^ + 0.0423 NADPH + 1.21 ATP + 0.0072 Rib5P + 0.0147 E4P + 0.044 OAA+ 0.063 αKG_mit_ + 0.0137 ACCoA_cyt_ => 1 C-mol Protein + 0.004 GA3P + 0.083 NADH + 0.0423 NADP^+^ + 1.21 ADP + 1.21 Pi + 0.0448 CO_2_

**2. Carbohydrate synthesis** (Composition derived [51]**)**

1. 0.113 Glc6P + 0.053 Fru6P + 0.167 ATP => 1 C-mol Carbohydrate + 0.167 ADP

**3. Lipids synthesis** (derived from the mean lipid composition from [30])

1. 0.002 Glc6P + 0.0055 Pyr + 0.011 GA3P + 0.006 CO_2_ + 0.039 ACCoA_mit_ + 0.441 ACCoA_cyt_ + 0.07 NADH + 0.599 NADPH + 0.42 ATP + 0.065 O_2_ => 1 C-mol Lipid + 0.07 NAD^+^ + 0.599 NADP^+^ + 0.42 ADP + 0.42 Pi

**4. RNA synthesis (derived from the RNA composition [57])**

1. 0.056 Pyr + 0.1136 CO_2_ + 0.105 Rib5P + 0.104 NAD+ + 0.075 NADPH + 1.1 ATP + 0.0479 OAA=> 1 C-mol RNA + 1.1 Pi + 1.1 ADP + 0.075 NADP^+^ + 0.104 NADH

**5. DNA synthesis (composition derived from [57] and the DNA polymerization [51])**

1. 0.051 Pyr +0.102 NAD^+^ + 0.273 NADPH + 1.146 ATP+ 0.132 CO_2_ + 0.102 Rib5P + 0.051 OAA => 1 C-mol DNA + 1.146 Pi + 1.146 ADP + 0.102 NADH + 0.273 NADP^+^

**1.2 Reactions and atom transitions network used in ^13^C-MFA, following the notation of [65].**

| **Name** |  | **Reaction** | |  |
| --- | --- | --- | --- | --- |
| **feedGlcB:** |  | FullyGlu > Glu_ext_ |  | |
|  |  | #abcdef > #abcdef |  | |
| **feedGlcC:** |  | CGlu > Glu_ext_ |  | |
|  |  | #abcdef > #abcdef |  | |
| **uptGlc:** |  | Glu_ext_ > Glu_int_ |  | |
|  |  | #abcdef > #abcdef |  | |
| **feedMeOHB:** |  | MetohL > Metoh_ext_ |  | |
|  |  | #a > #a |  | |
| **uptMeOH:** |  | Metoh_ext_ > Metoh_int_ |  | |
|  |  | #a > #a |  | |
| **upt1:** |  | Glu_int_ > Glc6P |  | |
|  |  | #abcdef > #abcdef |  | |
| **upt2:** |  | Metoh_int_ > Form |  | |
|  |  | #a > #a |  | |
| **TRE1:** |  | Glc6P > T6P |  | |
|  |  | #abcdef > #abcdef |  | |
| **TRE2:** |  | T6P > Treh |  | |
|  |  | #abcdef > #abcdef |  | |
| **TRE3:** |  | Treh > Glu_int_ |  | |
|  |  | #abcdef > #abcdef |  | |
| **emp1:** |  | Glc6P <> Fru6P |  | |
|  |  | #abcdef > #abcdef |  | |
| **emp2:** |  | Fru6P > FBP |  | |
|  |  | #abcdef > #abcdef |  | |
| **emp2B** |  | FBP > Fru6P |  | |
|  |  | #abcdef > #abcdef |  | |
| **emp3:** |  | FBP <> DHAP + GA3P |  | |
|  |  | #abcdef > #cba + #def |  | |
| **emp4:** |  | DHAP <> GA3P |  | |
|  |  | #abc > #abc |  | |
| **emp5:** |  | GA3P <> PG3 |  | |
|  |  | #abc > #abc |  | |
| **emp6:** |  | PG3 <> PG2 |  | |
|  |  | #abc > #abc |  | |
| **emp7:** |  | PG2 <> Pep |  | |
|  |  | #abc > # abc |  | |
| **emp8:** |  | Pep > Pyr |  | |
|  |  | #abc > #abc |  | |
| **emp9:** |  | Pyr > ACCoA_cyt_ + CO2 |  | |
|  |  | #abc > #bc + #a |  | |
| **emp10:** |  | Pyr + CO2 > OAA_mit_ |  | |
|  |  | #abc + #d > #abcd |  | |
| **emp11:** |  | Pyr > Pyrt |  | |
|  |  | #abc > #abc |  | |
| **emp11A:** |  | Pyr > Pyr_mit_ |  | |
|  |  | #abc > #abc |  | |
| **emp11B:** |  | Pyr_mit_ > Pyrt |  | |
|  |  | #abc > #abc |  | |
| **emp11C:** |  | NPyr > Pyr_mit_ |  | |
|  |  | #ABC > #ABC |  | |
| **emp11D:** |  | Pyr_mit_ > Pyr_ext_ |  | |
|  |  | #ABC > #ABC |  | |
| **emp12:** |  | ACCoA_cyt_ > ACCoA_mit_ |  | |
|  |  | #AB > #AB |  | |
| **ppp1:** |  | Glc6P > CO2 + Rul5P |  | |
|  |  | #abcdef > #a + #bcdef |  | |
| **ppp2:** |  | Rul5P <> Xul5P |  | |
|  |  | #abcde > #abcde |  | |
| **ppp3:** |  | Rul5P <> Rib5P |  | |
|  |  | #abcde > #abcde |  | |
| **ppp4:** |  | Xul5P + E4P <> GA3P + Fru6P |  | |
|  |  | #ABCDE + #abcd > #CDE + #ABabcd |  | |
| **ppp5:** |  | Xul5P + Rib5P <> Sed7P + GA3P |  | |
|  |  | #abcde + #ABCDE > #ABabcde + #CDE |  | |
| **ppp6:** |  | GA3P + Sed7P <> E4P + Fru6P |  | |
|  |  | #ABC + #abcdefg > #defg + #abcABC |  | |
| **TCA1:** |  | Pyr_mit_ > ACCoA_mit_ + CO2 |  | |
|  |  | #ABC > #BC + #A |  | |
| **TCA2:** |  | ACCoA_mit_ + OAA > CIT_mit_ |  | |
|  |  | #AB + #abcd > #dcbaBA |  | |
| **TCA3:** |  | CIT_mit_ > αKG + CO_2_ |  | |
|  |  | #ABCDEF > #ABCEF + #D |  | |
| **TCA4:** |  | αKG > SUCC + CO_2_ |  | |
|  |  | #ABCDE > #BCDE + #A |  | |
| **TCA4B:** |  | αKG > SUCC + CO_2_ |  | |
|  |  | #ABCDE > #EDCB + #A |  | |
| **TCA5:** |  | SUCC <> FUM |  | |
|  |  | #ABCD > #ABCD |  | |
| **TCA5B:** |  | SUCC <> FUM |  | |
|  |  | #ABCD > #DCBA |  | |
| **TCA6:** |  | FUM <> MAL |  | |
|  |  | #ABCD > #ABCD |  | |
| **TCA7:** |  | MAL <> OAA |  | |
|  |  | #ABCD > #ABCD |  | |
| **TCA8:** |  | Asp > FUM |  | |
|  |  | #ABCD > #ABCD |  | |
| **Met1:** |  | Form > CO_2_ |  | |
|  |  | #A > #A |  | |
| **Met2:** |  | Xul5P + Form > DHA + GA3P_per_ |  | |
|  |  | #ABCDE + #F > #FAB + #CDE |  | |
| **Met2B:** |  | Xul5P + Form > DHA + GA3P_per_ |  | |
|  |  | #ABCDE + #F > #ABF + #CDE |  | |
| **Met3:** |  | DHA > DHAP |  | |
|  |  | #ABC > #ABC |  | |
| **Met4:** |  | GA3P_per_ > GA3P |  | |
|  |  | #ABC > #ABC |  | |
| **BIO1:** |  | Glc6P > Glc6P_bio_ |  | |
|  |  | #ABCDEF > #ABCDEF |  | |
| **BIO2:** |  | Fru6P > Fru6P_bio_ |  | |
|  |  | #ABCDEF > #ABCDEF |  | |
| **BIO3:** |  | ACCoA_cyt_ > ACCoA_bio_ |  | |
|  |  | #BC > #BC |  | |
| **BIO4:** |  | OAA > OAA_bio_ |  | |
|  |  | #ABCD > #ABCD |  | |
| **BIO5:** |  | E4P > E4P_bio_ |  | |
|  |  | #ABCD > #ABCD |  | |
| **BIO6:** |  | Rib5P > Rib5P_bio_ |  | |
|  |  | #ABCDE > #ABCDE |  | |
| **BIO7:** |  | GA3P > GA3P_bio_ |  | |
|  |  | #ABC > #ABC |  | |
| **BIO8:** |  | αKG_mit_ > αKG_bio_ |  | |
|  |  | #ABCDE > #ABCDE |  | |
| **BIO9:** |  | Pyrt > Pyr_bio_ |  | |
|  |  | #ABC > #ABC |  | |
| **CO2out1:** |  | CO_2_ > CO2_ext |  | |
|  |  | #A > #A |  | |
| **aa_ala:** |  | Pyr <> Ala |  | |
|  |  | #ABC > #ABC |  | |
| **aa_glu:** |  | αKG_mit_ <> Glut |  | |
|  |  | #ABCDE > #ABCDE |  | |
| **aa_asp:** |  | OAA <> Asp |  | |
|  |  | #ABCD > #ABCD |  | |

**1.3 Reaction network used for anaNET analysis**

| **Abbreviation** | **Reaction** |
| --- | --- |
| HXK | Glc_int_ + ATP > Glc6P + ADP |
| PGI | Glc6P <> Fru6P |
| PFK | ATP + Fru6P > ADP + FBP + h |
| FB | FBP + h2o > Fru6P + Pi |
| FBA | FBP <> DHAP + GA3P |
| TPI | DHAP <> GA3P |
| GAPDH | GA3P + NAD + pi <> h + NADH + 13dpg |
| PGK | 13dpg + ADP <> PG3 + ATP |
| GPM | PG3 <> PG2 |
| ENO | PG2 <> h2o + Pep |
| PYK | ADP + h + Pep > ATP + Pyr |
| G6PDH | Glc6P + NADP > PG6 + h + NADPH |
| 6PGDH | PG6 + NADP + h2o > Rul5p + NADPH + h + CO_2_tot |
| RPI | Rib5p <> Rul5p |
| RPE | Rul5p <> Xul5p |
| TK(1)+TA | Rib5p + Xul5p <> Fru6P + E4P |
| TK(3) | E4P + Sed7P <> Fru6P + Rib5p |
| TA | GA3P + Sed7P <> Fru6P + E4P |
| TK(1) | Rib5p+ Xul5p <> GA3P + Sed7P |
| G3PDH | DHAP + NADH <> glyc3p + NAD |
| PYRCK | Pyr + ATP + CO_2_tot <> OAA + ADP + Pi + h2o |
| TPP | (2) Glc6P + utp + h2o > Treh + ppi + udp + Pi |
| TreP | Treh + h2o > (2) Glu_int_ |
| PDC | Pyr > ACALD + CO_2_tot |
| DHAK | DHA + ATP <> DHAP + ADP |
| PMI | Fru6P <> Man6p |
| CAT | Metoh + (0.5) o2 + (2) NAD > CO2tot + (2) NADH |
| MET | Metoh + Xul5p + (0.5) o2 > DHA + GA3P_per_ |
