# Supplementary material for: Glucose-methanol co-utilization in Pichia pastoris studied by metabolomics and instationary 13C flux analysis
Source: BMC Syst Biol. 2013 Feb 28;7:17. doi: 10.1186/1752-0509-7-17 (PMC3626722; doi:10.1186/1752-0509-7-17)
Supplement: Additional file 4 — Measured extra- and intracellular concentrations at steady-state (D = 0.09 h-1). [file 1752-0509-7-17-S4.docx]

**Additional file 4**. Measured extra- and intracellular metabolite concentrations at steady-state (D=0.09 h^-1^).

**Table 3.1. Measured intra- and extracellular amino acid concentrations**

| ***Amino acid*** | ***Extracellular  μmol/L*** | | ***Intracellular μmol/g_CDW_*** | |
| --- | --- | --- | --- | --- |
|  | ***value*** | ***sd*** | ***value*** | ***sd*** |
| **Ala** | 1.19 | 0.22 | 14.29 | 0.40 |
| **Gly** | 0.44 | 0.33 | 1.20 | 1.04 |
| **Val** | n.d. | n.d. | 1.24 | 0.04 |
| **Leu** | 0.06 | 0.03 | 0.63 | 0.02 |
| **Ile** | 0.04 | 0.02 | 0.28 | 0.02 |
| **Pro** | 0.03 | 0.03 | 2.57 | 0.04 |
| **Ser** | n.d | n.d. | 5.50 | 0.05 |
| **Thr** | 0.04 | 0.02 | 2.44 | 0.08 |
| **Asp** | 0.08 | 0.00 | 38.68 | 0.53 |
| **Phe** | 0.02 | 0.01 | 0.17 | 0.02 |
| **Orn** | 1.53 | 1.08 | 21.23 | 1.81 |
| **Glut** | n.d. | n.d. | 84.31 | 2.58 |
| **Lys** | 0.27 | 0.18 | 6.83 | 0.06 |
| **Asn** | 0.11 | 0.07 | 4.20 | 0.09 |
| **Gln** | 0.01 | 0.06 | 83.27 | 2.40 |
| **Tyr** | 0.03 | 0.02 | 0.18 | 0.02 |
| **His** | 0.01 | 0.02 | 4.15 | 0.09 |
| **Trp** | n.d. | n.d. | 0.08 | 0.01 |
| **Met** | n.d. | n.d. | 0.44 | 0.04 |

**Table 3.2. Measured intra- and extracellular metabolite concentrations.**

| **Metabolite** | ***Extracellular μmol/L*** | | ***Intracellular μmol/g_CDW_*** | |
| --- | --- | --- | --- | --- |
|  | ***value*** | ***sd*** | ***value*** | ***sd*** |
| **FUM** | 0.36 | 0.02 | 0.67 | 0.03 |
| **SUCC** | 0.27 | 0.04 | 1.79 | 0.09 |
| **MAL** | 0.09 | 0.11 | 2.49 | 2.16 |
| **αKG** | 0.47 | 0.03 | 1.27 | 0.08 |
| **Pep** | 0.03 | 0.05 | 0.66 | 0.02 |
| **Glu** | 35.77 | 3.99 | 0.78 | 0.86 |
| **GA3P** | n.d. | n.d. | 0.001 | 0.0001 |
| **Citrate** | n.d | n.d. | 7.03 | 0.20 |
| **PG2** | 0.05 | 0.01 | 0.20 | 0.02 |
| **IsoCitrate** | n.d. | n.d. | 0.05 | 0.00 |
| **DHAP** | n.d. | n.d. | 0.71 | 0.02 |
| **PG3** | 0.06 | 0.00 | 1.78 | 0.06 |
| **E4P** | 0.38 | 0.01 | 0.08 | 0.00 |
| **Rib5P** | 0.08 | 0.01 | 0.88 | 0.07 |
| **Rul5P** | n.d. | n.d. | 0.23 | 0.03 |
| **Xul5P** | n.d. | n.d. | 0.16 | 0.02 |
| **Man6P** | 0.14 | 0.04 | 1.16 | 0.03 |
| **Fru6P** | 0.17 | 0.02 | 3.19 | 0.13 |
| **Glc6P** | 0.52 | 0.05 | 14.31 | 0.43 |
| **Sed7P** | 0.27 | 0.02 | 5.41 | 0.17 |
| **FBP** | 0.21 | 0.13 | 0.93 | 0.05 |
| **T6P** | 0.12 | 0.00 | 0.11 | 0.01 |
| **Treh**  **Pyr** | 1.69  43.19 | 0.06  4.99 | 24.50  1.62 | 0.71  0.18 |
