# Supplementary material for: Glucose-methanol co-utilization in Pichia pastoris studied by metabolomics and instationary 13C flux analysis
Source: BMC Syst Biol. 2013 Feb 28;7:17. doi: 10.1186/1752-0509-7-17 (PMC3626722; doi:10.1186/1752-0509-7-17)
Supplement: Additional file 5 — Tables of the all Intracellular concentrations and Gibbs energy in P. pastoris cells growing on glucose-methanol chemostat. [file 1752-0509-7-17-S5.docx]

**Additional file 5.** Results from the ^13^C flux analysis: Estimated intracellular fluxes under methanol : glucose condition with the calculated standard deviations. The results are in mmol/gDCWh.

| ***flux*** | ***value*** | ***sd*** | ***flux*** | ***value*** | ***sd*** |
| --- | --- | --- | --- | --- | --- |
| aa_ala_bwd | 0.511 | 0.044 | feedGlcB_fwd | 0.141 | 0.001 |
| aa_ala_fwd | 0.511 | 0.044 | feedGlcC_fwd | 0.564 | 0.006 |
| aa_asp_bwd | 27.326 | 11.017 | feedMeOHB_fwd | 0.938 | 0.019 |
| aa_asp_fwd | 27.768 | 11.000 | Met1_fwd | 0.504 | 0.071 |
| aa_glu_bwd | 41.163 | 25.000 | Met2_fwd | 0.217 | 0.034 |
| aa_glu_fwd | 41.163 | 25.000 | Met2B_fwd | 0.217 | 0.034 |
| bio1_fwd | 0.110 | 0.005 | Met3_fwd | 0.434 | 0.068 |
| bio2_fwd | 0.052 | 0.002 | Met4_fwd | 0.434 | 0.068 |
| bio3_fwd | 0.070 | 0.034 | ppp1_fwd | 0.550 | 0.098 |
| bio4_fwd | 0.093 | 0.003 | ppp2_bwd | 0.217 | 0.061 |
| bio5_fwd | 0.028 | 0.001 | ppp2_fwd | 0.696 | 0.033 |
| bio6_fwd | 0.033 | 0.002 | ppp3_bwd | 0.000 | 0.064 |
| bio7_fwd | 0.008 | 0.000 | ppp3_fwd | 0.070 | 0.038 |
| bio8_fwd | 0.121 | 0.002 | ppp4_bwd | 1.665 | 0.168 |
| bio9_fwd | 0.295 | 0.008 | ppp4_fwd | 1.674 | 0.160 |
| CO2out1_fwd | 1.904 | 0.104 | ppp5_bwd | 0.077 | 0.140 |
| emp1_bwd | 1.102 | 0.066 | ppp5_fwd | 0.114 | 0.130 |
| emp1_fwd | 1.148 | 0.072 | ppp6_bwd | 1.264 | 0.077 |
| emp10_wd | 0.214 | 0.004 | ppp6_fwd | 1.301 | 0.057 |
| emp11_fwd | 0.001 | 0.012 | Tca1_fwd | 0.247 | 0.055 |
| emp11A_fwd | 0.541 | 0.056 | Tca2_fwd | 0.372 | 0.069 |
| emp11B_fwd | 0.293 | 0.015 | Tca3_fwd | 0.372 | 0.069 |
| emp11C_fwd | 1.087 | 0.072 | Tca4_fwd | 0.125 | 0.035 |
| emp11D_fwd | 1.087 | 0.072 | Tca4B_fwd | 0.125 | 0.035 |
| emp12_fwd | 0.124 | 0.044 | Tca5_bwd | 0.188 | 0.066 |
| emp2_fwd | 0.186 | 0.020 | Tca5_fwd | 0.313 | 0.056 |
| emp2B_fwd | 0.145 | 0.024 | Tca5B_fwd | 0.313 | 0.056 |
| emp3_bwd | 1.650 | 0.621 | Tca5B_bwd | 0.188 | 0.066 |
| emp3_fwd | 1.691 | 0.620 | Tca6_bwd | 0.993 | 1.352 |
| emp4_bwd | 96.980 | 150.000 | Tca6_fwd | 1.257 | 1.200 |
| emp4_fwd | 97.454 | 150.000 | Tca7_bwd | 6.018 | 7.028 |
| emp5_bwd | 3.750 | 4.900 | Tca7_fwd | 6.282 | 7.010 |
| emp5_fwd | 4.700 | 4.900 | Tca8_fwd | 0.013 | 0.021 |
| emp6_bwd | 0.995 | 3.300 | TRE1_fwd | 0.010 | 0.016 |
| emp6_fwd | 1.945 | 3.300 | TRE2_fwd | 0.010 | 0.016 |
| emp7_bwd | 0.005 | 0.096 | TRE3_fwd | 0.010 | 0.016 |
| emp7_fwd | 0.955 | 0.079 | upt1 | 0.716 | 0.001 |
| emp8_fwd | 0.950 | 0.054 | upt2 | 0.938 | 0.019 |
| emp9_fwd | 0.194 | 0.009 | uptGlc | 0.705 | 0.006 |
|  |  |  | uptMeOH | 0.938 | 0.019 |

| **Estimated parameters (μmol/gDCW)** | | | | | |
| --- | --- | --- | --- | --- | --- |
| **Metabolites** | **value** | **sd** | **Metabolites** | **value** | **sd** |
| Form | 2.86 | 1.9 | Metoh_ext_ | 1.414 | 2.3 |
| GAP | 0.0047 | 1.1 | Metoh_int_ | 0.1355 | 1.2 |
| DHA | 0.109 | 0.55 | OAA | 0.1165 | 2.9 |
| ACCoA_mit_ | 0.48 | 5 | CO2 | 0.0338 | 7.3 |
