# Supplementary material for: Glucose-methanol co-utilization in Pichia pastoris studied by metabolomics and instationary 13C flux analysis
Source: BMC Syst Biol. 2013 Feb 28;7:17. doi: 10.1186/1752-0509-7-17 (PMC3626722; doi:10.1186/1752-0509-7-17)
Supplement: Additional file 6 — Results from the INST 13C flux analysis. Estimated intracellular fluxes under methanol-glucose condition with the calculated standard deviations. The network description can be found in Additional file 2 (1.1). [file 1752-0509-7-17-S6.docx]

***Additional file 6.***

***Table 1.***Intracellular concentrations of coenzyme and nucleotides pools in P. pastoris cells growing on glucose:methanol. Concentration are given in μmol/g_DCW_

| **Intracellular Concentratrions (μmol/g_DCW_)** | | |
| --- | --- | --- |
| **Coenzymes** | **Value** | **SD** |
| **NAD+NADH** | 7.470 | 0.18 |
| **NADP+NADPH** | 0.180 | 0.03 |
| **Acetyl CoA** | 0.18 | 0.03 |
| **FAD** | 0.73 | 0.12 |

| **Intracellular Concentratrions (μmol/g_DCW_)** | | |
| --- | --- | --- |
| **Nucleotides** | **Value** | **SD** |
| **ATP** | 6.57 | 0.15 |
| **ADP** | 0.94 | 0.03 |
| **GTP** | 0.99 | 0.04 |
| **GMP** | 0.72 | 0.11 |
| **AMP** | 0.29 | 0.01 |
| **GDP** | 0.19 | 0.01 |
| **cAMP** | 0.01 | 0.00 |

***Table 2***. Intacellular concentrations of central metabolite pools in P. Pastoris cells growing on glucose:methanol. Concentrations are given in μmol/g_DCW._

| **Intracellular Concentrations μmol/g_DCW_)** | | | | | | | |
| --- | --- | --- | --- | --- | --- | --- | --- |
|  | **Experiments 1** | | **Experiments 2** | | **Average values** | |  |
| **Metabolites** | **Value** | **SD** | **Value** | **SD** | **Value** | **SD** |  |
| **Treh** | 24.503 | 0.706 | n.d. | n.d. | 24.503 | 0.706 |  |
| **Glc6P** | 14.315 | 0.431 | 14.570 | 0.291 | 14.442 | 0.550 |  |
| **CIT** | 7.032 | 0.200 | 7.299 | 0.150 | 7.165 | 0.250 |  |
| **Sed7P** | 5.413 | 0.165 | 5.371 | 0.116 | 5.392 | 0.204 |  |
| **Fru6P** | 3.187 | 0.134 | 3.105 | 0.020 | 3.146 | 0.147 |  |
| **MAL** | 2.489 | 2.156 | 3.187 | 0.324 | 2.838 | 2.236 |  |
| **SUCC** | 1.790 | 0.087 | 1.886 | 0.120 | 1.838 | 0.148 |  |
| **αKG** | 1.274 | 0.078 | 2.119 | 0.176 | 1.696 | 0.192 |  |
| **PG3** | 1.781 | 0.057 | 1.955 | 0.082 | 1.868 | 0.100 |  |
| **Pyr** | 1.617 | 0.182 | 1.331 | 0.078 | 1.474 | 0.198 |  |
| **Pep** | 0.657 | 0.024 | 0.755 | 0.043 | 0.706 | 0.049 |  |
| **Glu** | 0.784 | 0.862 | n.d. | n.d. | 0.784 | 0.862 |  |
| **GA3P** | 0.005 | 0.001 | n.d. | n.d. | 0.005 | 0.001 |  |
| **PG2** | 0.197 | 0.015 | 0.102 | 0.060 | 0.149 | 0.062 |  |
| **ICIT** | 0.046 | 0.002 | 0.024 | 0.028 | 0.035 | 0.032 |  |
| **DHAP** | 0.711 | 0.017 | n.d. | n.d. | 0.711 | 0.017 |  |
| **E4P** | 0.083 | 0.003 | n.d. | n.d. | 0.083 | 0.003 |  |
| **Rib5P** | 0.883 | 0.065 | n.d. | n.d. | 0.883 | 0.065 |  |
| **Rul5P** | 0.228 | 0.031 | n.d. | n.d. | 0.228 | 0.031 |  |
| **Xul5P** | 0.160 | 0.017 | n.d. | n.d. | 0.160 | 0.017 |  |
| **Man6P** | 1.162 | 0.026 | 1.284 | 0.016 | 1.223 | 0.030 |  |
| **FUM** | 0.667 | 0.027 | 0.872 | 0.034 | 0.770 | 0.044 |  |
| **FBP** | 0.933 | 0.051 | 0.888 | 0.034 | 0.910 | 0.061 |  |
| **T6P** | 0.105 | 0.007 | 0.083 | 0.010 | 0.094 | 0.012 |  |

***Table 3***. Intracellular concentrations of amino acid pools in P. Pastoris cells growing on glucose:methanol. Concentrations are given in μmol/g_DCW._

| ***Intracellular Concentrations*** | | | | | | |
| --- | --- | --- | --- | --- | --- | --- |
|  | ***Experiments 1*** | | ***Experiments 2*** | | ***Average values*** | |
| ***Amino acids*** | **Value** | **SD** | **Value** | **SD** | **Value** | **SD** |
| **Glut** | 84.31 | 2.58 | 85.40 | 1.47 | 84.85 | 2.97 |
| **Gln** | 83.27 | 2.40 | 86.67 | 0.04 | 84.97 | 2.40 |
| **Asp** | 38.68 | 0.53 | 40.43 | 0.16 | 39.56 | 0.56 |
| **Orn** | 21.23 | 1.81 | 23.95 | 0.45 | 22.59 | 1.87 |
| **Ala** | 14.29 | 0.40 | 15.73 | 0.63 | 15.01 | 0.75 |
| **Lys** | 6.83 | 0.06 | 13.61 | 0.12 | 10.22 | 0.13 |
| **Ser** | 5.50 | 0.05 | 6.39 | 0.09 | 5.94 | 0.10 |
| **Asn** | 4.20 | 0.09 | 5.12 | 0.04 | 4.66 | 0.10 |
| **His** | 4.15 | 0.09 | 5.43 | 0.12 | 4.79 | 0.15 |
| **Gly** | 1.20 | 1.04 | 1.46 | 0.68 | 1.33 | 1.24 |
| **Val** | 1.24 | 0.04 | 1.35 | 0.08 | 1.30 | 0.09 |
| **Leu** | 0.63 | 0.02 | 0.75 | 0.23 | 0.69 | 0.23 |
| **ile** | 0.28 | 0.02 | 0.37 | 0.05 | 0.33 | 0.06 |
| **Pro** | 2.57 | 0.04 | 2.65 | 0.06 | 2.61 | 0.07 |
| **Thr** | 2.44 | 0.08 | 2.54 | 0.14 | 2.49 | 0.16 |
| **Phe** | 0.17 | 0.02 | 0.24 | 0.05 | 0.20 | 0.06 |
| **Tyr** | 0.18 | 0.02 | 0.23 | 0.03 | 0.20 | 0.04 |
| **Trp** | 0.08 | 0.01 | 0.10 | 0.03 | 0.09 | 0.03 |
| **Met** | 0.44 | 0.04 | 0.53 | 0.03 | 0.48 | 0.05 |

***Table 4***.Maximun gibbs energy at a minimun substrate and minimun gibbs energy at a máximum subsrate of P. Pastoris growing on glucose:methanol.

| **Gibbs energy (kJ/mol)** | | |
| --- | --- | --- |
| **Reaction** | **Δ Gibbs Min.** | **Δ Gibbs Max.** |
| HXK | -26.08 | -12.81 |
| PGI | -0.67 | -0.43 |
| PFK | -33.06 | -30.43 |
| FB | -18.88 | -16.85 |
| FBA | -10.74 | -9.93 |
| TPI | -5.17 | -4.54 |
| GAPDH | -2.31 | 0.00 |
| PGK | -2.31 | 0.00 |
| GPM | -1.22 | 0.00 |
| ENO | -0.91 | -0.54 |
| PYK | -33.29 | -19.82 |
| G6PDH | -27.81 | 0.00 |
| 6PGDH | -226.33 | -190.21 |
| RPI | -3.08 | -2.72 |
| RPE | -2.40 | -2.00 |
| TK(1)+TA | -0.15 | 0.00 |
| TK(3) | -12.63 | -12.25 |
| TA | 6.15 | 6.95 |
| TK(1) | -6.95 | -6.15 |
| G3PDH | -32.54 | -13.02 |
| PYRCK | -164.34 | -139.58 |
| TPP | -60.61 | -29.77 |
| TreP | -58.47 | -36.67 |
| PDC | -209.69 | -177.54 |
| DHAK | -28.01 | -11.67 |
| PMI | -1.71 | -1.50 |
| CAT | -466.06 | -432.40 |
| MET | -77.45 | -39.47 |
